# Choroidal vascular changes in early-stage myopic maculopathy from deep learning choroidal analysis: a hospital-based SS-OCT study

**DOI:** 10.1186/s40662-024-00398-x

**Published:** 2024-08-06

**Authors:** Yan Li, Haoer Li, Xue Rui, Yuan Wang, Shenju Zhu, Mengge Huang, Jianqiang Liang, Yangfeifei Zhu, Jiajia Shi, Le Yu, Shenghai Huang, Chun Yang, Mengmeng Dong, Hebei Gao, Meixiao Shen, Hao Wu, Xiangtian Zhou

**Affiliations:** 1 National Clinical Research Center for Ocular Diseases, Eye Hospital, Wenzhou Medical University, Wenzhou, 325027 China; 2 State Key Laboratory of Ophthalmology, Optometry and Visual Science, Eye Hospital, Wenzhou Medical University, Wenzhou, 325027 China; 3 Oujiang Laboratory (Zhejiang Lab for Regenerative Medicine, Vision, and Brain Health), Eye Hospital, Wenzhou Medical University, Wenzhou, 325035 Zhejiang China; 4 Research Unit of Myopia Basic Research and Clinical Prevention and Control, Chinese Academy of Medical Sciences (2019RU025), Wenzhou, Zhejiang China; 5 National Engineering Research Center of Ophthalmology and Optometry, Eye Hospital, Wenzhou Medical University, Wenzhou, 325027 China; 6 School of Artificial Intelligence, Wenzhou Polytechnic, Wenzhou, 325035 China

**Keywords:** High myopia, Myopic maculopathy, Diffuse chorioretinal atrophy, Choroidal vasculature, Deep learning

## Abstract

**Background:**

The objective of this study is to illustrate the changes in the choroidal vasculature in individuals with diffuse chorioretinal atrophy (DCA, early-stage myopic maculopathy) and investigate the association between them.

**Methods:**

This study included 1418 highly myopic eyes from 720 participants aged 18 − 60 years from the Wenzhou High Myopia Cohort Study. These participants underwent comprehensive ophthalmic assessments. Myopic maculopathy classification followed the Meta-PM system, with pathological myopia defined as myopic maculopathy of DCA or severer. Eyes with myopic maculopathy categorized as no macular lesions (C0), tessellated fundus (C1), and DCA (C2) were enrolled in the analysis. Choroidal images were obtained from swept-source optical coherence tomography (SS-OCT), and the images were processed with a deep learning-based automatic segmentation algorithm and the Niblack auto-local threshold algorithm.

**Results:**

DCA was detected in 247 eyes (17.4%). In comparison to eyes with C0, those with C2 exhibited significant reductions in choroidal thickness (ChT), luminal area (LA), and stromal area (SA) across all evaluated regions (all *P* < 0.001). An increase in choroidal vascular index (CVI) was observed in all regions, except for the nasal perifoveal (N2) and inferior perifoveal (I2) regions (all *P* < 0.01). Multivariable logistic regression analysis revealed a negative association between the presence of DCA and increases in choroidal LA and SA (odds ratio ≤ 0.099, *P* < 0.001). Multivariable linear regression analysis showed that the mean deviation of the visual field test was positively associated with LA and SA at the vertical meridian (B = 1.512, *P* < 0.001 for LA; B = 1.956, *P* < 0.001 for SA). Furthermore, the receiver operating characteristic curve analyses showed the optimal ChT to diagnose pathological myopia was 82.4 µm in the N2 region, the LA was 0.076 mm^2^ and the SA was 0.049 mm^2^, with area under the curves of 0.916, 0.908, and 0.895, respectively.

**Conclusions:**

The results of this study indicated that both the presence of DCA and visual function impairment were associated with reductions in choroidal perfusion and stromal components. Moreover, we established threshold values for choroidal parameters in diagnosing pathological myopia, offering valuable references for clinical diagnosis and management.

**Supplementary Information:**

The online version contains supplementary material available at 10.1186/s40662-024-00398-x.

## Background

Recent decades have witnessed a significant surge in myopia prevalence worldwide, particularly in East and Southeast Asia. Alongside this trend, the prevalence of high myopia is also on the rise, expected to increase from 5.2% in 2020 to 9.8% by 2050 [1]. Highly myopic eyes can experience a variety of fundus complications, such as myopic maculopathies [2], development of glaucomatous or glaucoma-like optic neuropathy [3], and peripheral retinal degenerations, holes, and detachments [4, 5]. Among these, myopic maculopathy has emerged as a major cause of low vision or blindness globally and was recognized as the second leading cause of low vision in China [6–8]. Given its impact, the prevention and management of myopic maculopathy are key challenges in the field of high myopia research.

According to the classification system proposed by Ohno-Matsui et al. [9] and developed by Ruiz-Medrano et al. [2], myopic maculopathy can be categorized based on three key factors: atrophy, traction, and neovascularization. However, there is no treatment available for atrophic lesions, making it crucial to fully understand their histopathological characteristics. Diffuse chorioretinal atrophy (DCA) represents an early stage of atrophic myopic maculopathy, which also marks the onset of pathological myopia [10, 11]. It progresses with a notably high rate of 50% over four years and impairs visual function gradually [12, 13]. A deep understanding of the pathogenesis of DCA would help identify early intervention methods for myopic maculopathy.

Previous studies have found that older age, longer axial length (AL), and a higher degree of myopia were associated with the development of DCA [12, 14, 15]. Apart from these factors, as the primary blood supply source for the retina, the choroid has been reported to gradually thin as the severity of myopic maculopathy increases [16]. Studies on highly myopic children showed a relationship between the thinner choroidal thickness (ChT) and the presence of DCA [17, 18]. However, since ChT reflects the aggregate characteristics of all its components [19, 20], merely observing changes in its thickness is not adequate for fully understanding the pathogenesis of DCA. Elucidating the changes in the choroidal vessels and extravascular stroma and their relationship with DCA can prove beneficial.

Thus, we conducted this study to clarify the changes in the choroidal vasculature in individuals with early-stage myopic maculopathy, explore the correlation between the presence of DCA and the choroidal vasculature, as well as the impact of the choroidal vasculature on visual function. This study will also determine the cut-off values of choroidal parameters for diagnosing pathological myopia across different age groups and provide references for the diagnosis of pathological myopia in clinical practice.

## Methods

### Study design and participants

The current study was a hospital-based cross-sectional study. A subset of participants aged 18–60 years at the baseline examination of the Wenzhou High Myopia Cohort Study from November 2021 to September 2023 were included in the current study. We analyzed the baseline data of these individuals in this study.

The Wenzhou High Myopia Cohort Study started in November 2021, recruiting subjects aged 10–60 years from the optometry clinic. High myopia was defined as the spherical equivalent refractive (SER) ≤  − 6.00 D as measured by autorefractor after cycloplegia, or AL ≥ 26.5 mm. The inclusion and exclusion criteria are listed in Additional file 1: Table S1. The eligible participants will be followed up annually, over a period of 10 years. All examinations were carried out in the afternoon at the National Clinical Research Center for Ocular Diseases in the Eye Hospital of Wenzhou Medical University. All OCT images were taken by the same investigator. Investigators and technicians involved in examinations were trained technically and ethically to perform the study. The protocol of this study was approved by the institutional research ethics committee of the Eye Hospital of Wenzhou Medical University (2020–199-K-181) and registered at the Chinese Clinical Trial Registry (ChiCTR2100047424). All subjects provided a written informed consent for study participation, and the study adhered to the tenets of the Declaration of Helsinki.

### Examinations

All participants received detailed ophthalmic assessments, including measurements of best-corrected visual acuity (BCVA), AL, anterior chamber depth (ACD), central corneal thickness (CCT), lens thickness (LT), and intraocular pressure (IOP). The visual field test was carried out with a Humphrey Field Analyzer 750i (Carl Zeiss Meditec, USA) using central 24–2 program and Swedish Interactive Threshold Algorithm Fast (SITA-Fast) strategy. Visual field test with fixation loss < 20%, false positive errors < 15%, and false negative errors < 15% were included in the analysis of visual function. Fundus photography was performed with a digital retinal camera (Zeiss Visucam 224, Germany) after cycloplegia with tropicamide 0.5% and phenylephrine 0.5%. Two regions of each eye were photographed, with one centered on the optic disc and another centered on the macula.

### Grading of myopic maculopathy

Based on the META–PM classification system, myopic maculopathy was categorized into category 0 (C0, no macular lesions), category 1 [C1, tessellated fundus (TF)], category 2 (C2, DCA), category 3 (C3, patchy chorioretinal atrophy), category 4 (C4, macular atrophy) and plus lesions (lacquer cracks, choroidal neovascularization, and Fuchs spot) [9]. Detailed fundus grading was performed by three experienced examiners (YL, XR, YW) and questionable cases were evaluated by a retinal specialist (SH). The intergrader agreement between examiners was assessed using AC1 statistics in a subset of 100 highly myopic eyes (AC1, 0.96). Eyes with myopic maculopathy categorized as no macular lesions (C0), tessellated fundus (C1) and DCA (C2) were subject to analyses.

### SS-OCT image acquisition and analysis of choroidal parameter

The swept-source optical coherence tomography (SS-OCT) system (VG200D, SVision Imaging, Henan, China) containing a SS laser with a central wavelength of approximately 1050 nm and a scan rate of 200,000 A-scans per second was used to obtain choroidal images. A 12-mm radial line scan was performed to obtain two transverse section images (vertical and horizontal meridians) across the fovea. As individuals with high myopia are prone to reflex at the edge of images, 6-mm cross-sections of the macula that were centered on the fovea were selected for further analysis.

**Fig. 1:**
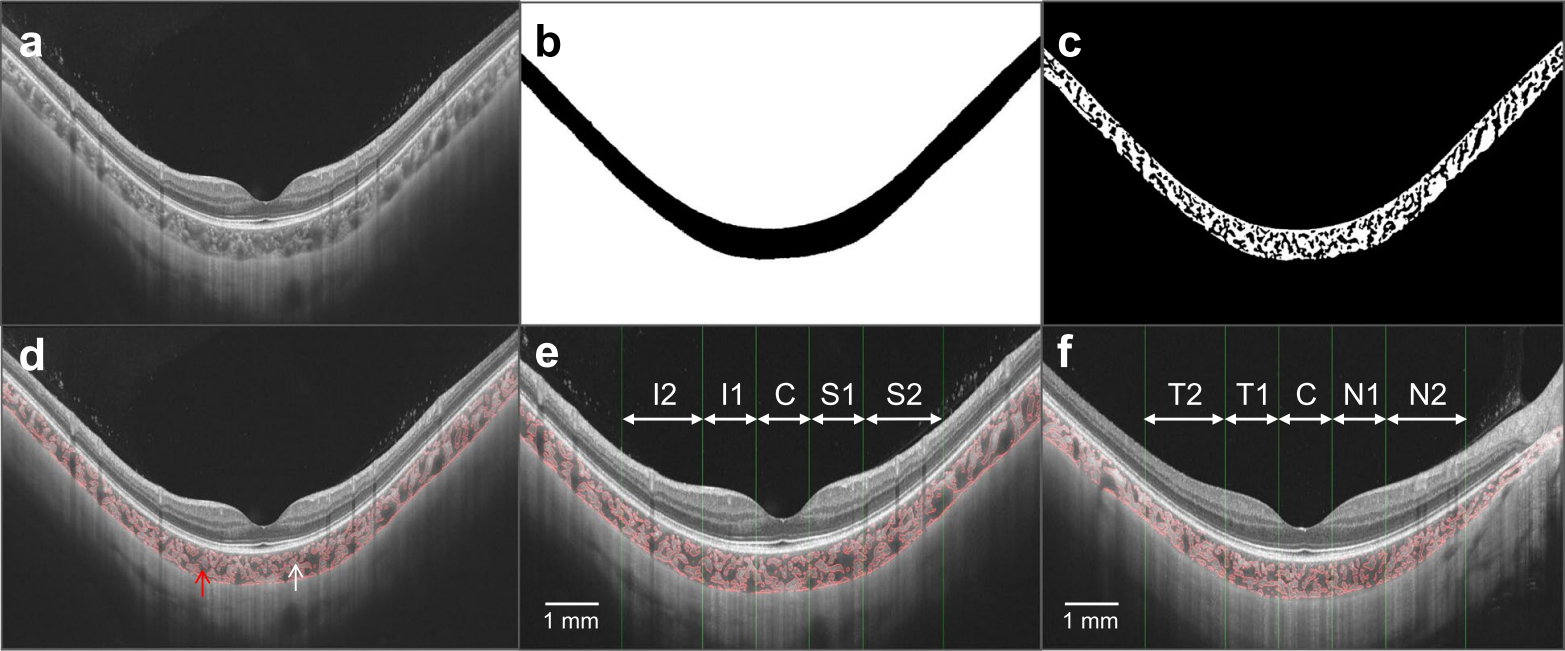
Illustration of choroidal image binarization and choroidal vascular parameters analysis. **a** The original swept-source optical coherence tomography (SS-OCT) image. **b** Segmentation of the choroidal boundaries. **c** Binarization of the choroidal area. **d** Overlay of the region after performing image binarization. **e** Vertical scan. **f** Horizontal scan. Regions: C, fovea; I1, inferior parafovea; I2, inferior perifovea; S1, superior parafovea; S2, superior perifovea; T1, temporal parafovea; T2, temporal perifovea; N1, nasal parafovea; N2, nasal perifovea. The choroidal thickness (ChT), luminal area (LA), stromal area (SA), and choroidal vascular index (CVI) in a 6-mm submacular region centered on the fovea were analyzed. Mean values of choroidal parameters at the vertical and horizontal meridians as well as in each region were respectively calculated. The low-reflection areas indicated by the red arrow are the vascular lumens while the high-reflection areas indicated by the white arrow are the extravascular stroma

A custom deep learning algorithm based on the improved residual U-Net architecture was used to automatically segment the choroid boundaries of all images. This neural network model was trained based on OCT images of 382 emmetropic and 217 highly myopic eyes, with an accuracy rate of 95% for correctly identifying choroidal boundaries. The results of choroidal boundary segmentation were checked twice. For images with segmentation errors, manual corrections were applied. Additionally, due to the impact of parapapillary atrophy (PPA) on the accuracy of choroidal boundary segmentation, images with severe PPA underwent manual segmentation. ChT was determined by measuring the distance between the choroidal boundaries. Subsequently, the luminal area (LA) and stromal area (SA) within the choroid were precisely defined. This precision was achieved through the application of Niblack auto-local threshold algorithm on binarized OCT images. The choroidal vascular index (CVI) was calculated as the ratio of the LA to the total choroidal area (TCA, LA + SA). Choroidal parameters were assessed across ten specific regions as depicted in Fig. 1.

We used Bennett’s formula, *t* = *p* × *q* × *s* (*t* as the real scan length, *p* as the magnification factor determined by the OCT imaging system camera, *q* as the magnification factor related to the eye, *s* as the original measurement from the OCT image), to adjust the image magnification based on the AL [21]. The correction factor *q* was determined by the formula *q* = 0.01306 × (AL – 1.82). When imaging the eye with an AL of 24.4 mm, the actual scanning range *t* would be equal to the *s*. When imaging eyes with other ALs, the real scan length *t* was determined by the equation *t* = (AL – 1.82) / 22.58 × *s.*

### Statistical analysis

Both eyes of an individual were included in the statistical analysis. Shapiro–Wilk tests were performed to determine the normality of the data distribution. Continuous and categorical data were presented as means ± SD and numbers with percentages, respectively. Characteristics were compared using χ^2^ tests for categorical parameters and generalized estimating equations (GEE) for continuous parameters. Bonferroni adjustments for multiple comparisons were applied to pairwise comparisons. In the regional analysis of choroidal parameters, the values of LA and SA in perifoveal regions were divided by 1.5 to eliminate the effects of inconsistent widths in each region. Multivariable logistic regression models were used to assess factors associated with the presence of DCA. In the multivariable logistic regression analysis, all continuous variables were subjected to Z-score standardization. Multivariable linear regression models were used to assess the association between visual function and choroidal parameters. Receiver operating characteristic (ROC) curve analysis was used to determine the optimal ChT, LA, and SA for each location to diagnose pathological myopia. A *P* value less than 0.05 was considered significant. All data were analyzed with SPSS v.26 and GraphPad Prism 9.

## Results

### General characteristics of participants

In the initial assessment of 811 individuals, 723 participants (1446 eyes) aged 18–60 years were eligible for study inclusion. We further checked for image quality, fundus pathological changes affecting the analysis of choroidal parameters, and categorization of myopic maculopathy. The analysis ultimately incorporated data from 706 right eyes and 712 left eyes of 720 participants. The exclusion of 17 right eyes was due to choroiditis (n = 2), polypoidal choroidal vasculopathy (n = 1), myopic maculopathy severer than C3 or the presence of “plus lesions” (n = 8), and focal choroidal excavation or peripapillary intrachoroidal cavitation (FCE or PICC, n = 8). Similarly, 11 left eyes were excluded due to choroiditis (n = 1), long-standing retinal detachment (n = 1), myopic maculopathy severer than C3 or the presence of “plus lesions” (n = 5), and FCE or PICC (n = 5).

The general characteristics among eyes with no macular lesions (C0), tessellated fundus (C1) and DCA (C2) were compared (Table 1). C0 was observed in 94 eyes (6.6%), C1 in 1077 eyes (76.0%), and C2 in 247 eyes (17.4%). The mean AL was 26.33 ± 1.02 mm in the C0 group, 26.79 ± 1.02 mm in the C1 group, and 27.90 ± 1.20 mm in the C2 group. The mean SER was − 7.37 ± 1.23 D in the C0 group, − 8.06 ± 1.69 D in the C1 group, and − 10.11 ± 2.69 D in the C2 group. There were significant differences among the three groups and between any one of the groups compared to the other two groups in AL and SER (all *P* < 0.001). There were no significant differences among three groups with respect to gender, weight, blood pressure, ACD and IOP. Compared with the C1 group, the individuals in the C2 group were older, had lower height, thicker lenses, and worse BCVA and visual function [*P* < 0.001 for age, BCVA, visual field index (VFI), mean deviation (MD), pattern standard deviation (PSD); *P* = 0.023 for height and LT]. However, there were no significant differences between the C1 and C0 groups in age, height, LT, BCVA, MD and PSD.

**Table 1:** Comparisons of general characteristics among highly myopic eyes with C0, C1 and C2

Characteristics	No macular lesions (C0)	Tessellated fundus (C1)	Diffuse chorioretinal atrophy (C2)	*P* value (All)	*P* value (C2–C1)	*P* value (C2–C0)	*P* value (C1–C0)
**Participant characteristics**							
No. of eyes (%)	94 (6.6)	1077 (76.0)	247 (17.4)				
Age (years)	32.8 ± 10.4	35.1 ± 9.6	39.1 ± 10.1	< 0.001^*^	< 0.001	< 0.001	0.097
Gender (male/female)	23 / 71	339 / 738	70 / 177	0.267^†^	> 0.05	> 0.05	> 0.05
Height (cm)	162.7 ± 9.3	162.5 ± 8.1	161.0 ± 7.6	0.025^*^	0.023	0.360	1.000
Weight (kg)	62.5 ± 12.5	59.8 ± 10.8	59.9 ± 9.5	0.138^*^	1.000	0.209	0.142
SBP (mmHg)	116.5 ± 13.4	114.4 ± 14.1	114.7 ± 15.2	0.329^*^	1.000	0.875	0.415
DBP (mmHg)	68.0 ± 10	66.8 ± 10.1	67.7 ± 10.9	0.279^*^	0.676	1.000	0.723
**Ocular biological parameters**							
No. of eyes (%)	94 (6.6)	1077 (76.0)	247 (17.4)				
AL (mm)	26.33 ± 1.02	26.79 ± 1.02	27.90 ± 1.20	< 0.001^*^	< 0.001	< 0.001	< 0.001
CCT (μm)	545 ± 32	540 ± 31	545 ± 33	0.037^*^	0.093	1.000	0.304
ACD (mm)	3.63 ± 0.31	3.57 ± 0.29	3.56 ± 0.27	0.165^*^	1.000	0.199	0.207
LT (mm)	3.84 ± 0.33	3.87 ± 0.36	3.94 ± 0.35	0.012^*^	0.023	0.044	1.000
Corneal curvature (D)	44.1 ± 1.6	43.8 ± 1.4	43.6 ± 1.5	0.029^*^	0.149	0.039	0.348
IOP (mmHg)	15.0 ± 2.6	14.4 ± 2.8	14.5 ± 2.8	0.127^*^	1.000	0.334	0.127
SER (D)	− 7.37 ± 1.23	− 8.06 ± 1.69	− 10.11 ± 2.69	< 0.001^*^	< 0.001	< 0.001	< 0.001
BCVA (logMAR)	− 0.028 ± 0.055	− 0.028 ± 0.056	− 0.007 ± 0.077	< 0.001^*^	< 0.001	0.012	1.000
**Visual function**							
No. of eyes (%)	69 (5.6)	942 (77.1)	211 (17.3)				
VFI (%)	97 ± 2	96 ± 5	94 ± 6	< 0.001^*^	< 0.001	< 0.001	0.044
MD (dB)	− 3.13 ± 1.73	− 3.48 ± 2.23	− 4.92 ± 2.61	< 0.001^*^	< 0.001	< 0.001	0.430
PSD (dB)	2.18 ± 1.42	2.32 ± 1.61	3.40 ± 1.93	< 0.001^*^	< 0.001	< 0.001	1.000

Data presented are mean ± SDs. Visual field test with fixation loss < 20%, false positive errors and false negative errors < 15% were included in the analysis of visual function

*SBP *= systolic blood pressure; *DBP *= diastolic blood pressure; *AL *= axial length; *CCT *= central corneal thickness; *ACD *= anterior chamber depth; *LT *= lens thickness; *IOP *= intraocular pressure; *SER *= spherical equivalent refractive; *LogMAR *= logarithm of the minimum angle of resolution; *VFI *= visual field index; *MD *= mean deviation; *PSD *= pattern standard deviation

^*^P values were determined by GEE, and Bonferroni adjustments for multiple comparisons were applied to pairwise comparisons. ^†^*P* values were determined by Pearson’s χ^2^ test, and Bonferroni adjustments for multiple comparisons were applied to pairwise comparisons

### Global and regional characteristics of choroidal vasculature among different types of myopic maculopathy

**Fig. 2:**
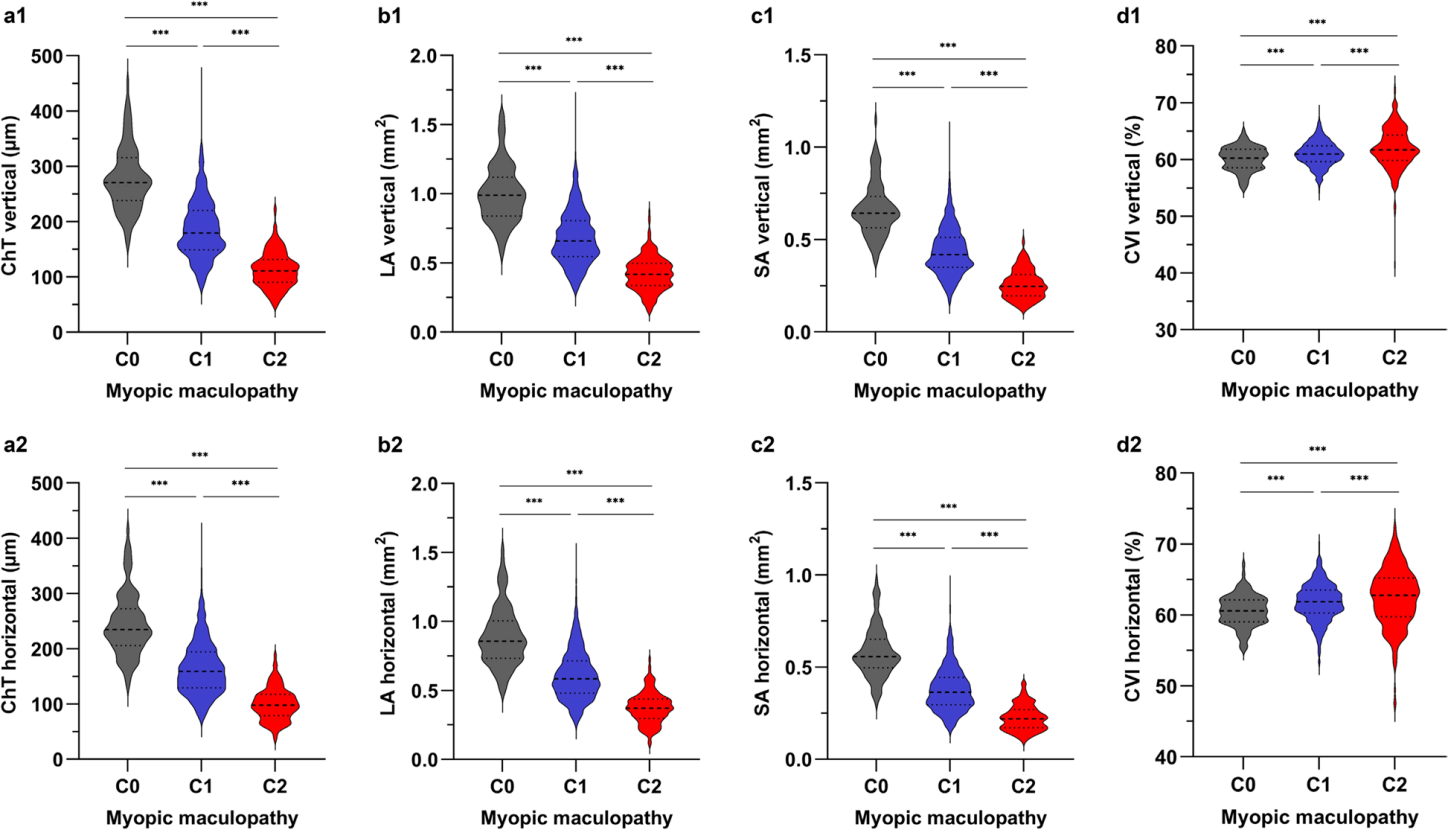
Violin plots comparing the global choroidal vascular parameters among the C0, C1 and C2 groups. **a1** ChT at the vertical meridian; **b1** LA at the vertical meridian; **c1** SA at the vertical meridian; **d1** CVI at the vertical meridian; **a2** ChT at the horizontal meridian; **b2** LA at the horizontal meridian; **c2** SA at the horizontal meridian; **d2** CVI at the horizontal meridian. The median was represented by the middle line within each violin, and the lower and upper quartiles were represented by the lower and upper lines within each violin, respectively. C0, no macular lesions; C1, tessellated fundus; C2, diffuse chorioretinal atrophy; ChT, choroidal thickness; LA, luminal area; SA, stromal area; CVI, choroidal vascular index. n = 94, 1077 and 247 eyes in the C0, C1 and C2 groups, respectively. The level of significance was detected with GEE adjusted for age, AL and the correlation between both eyes of an individual, ^***^P < 0.001

**Fig. 3:**
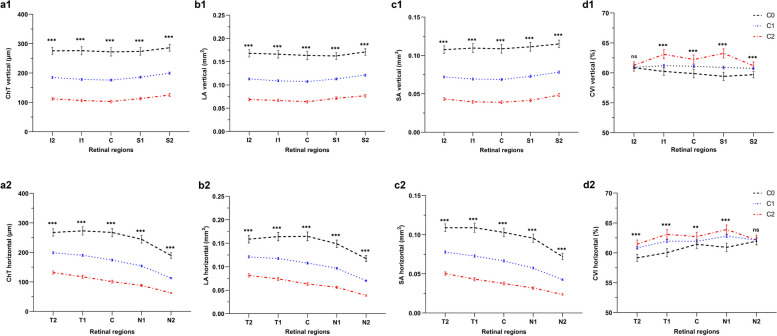
Regional analysis of choroidal vascular parameters among the C0, C1 and C2 groups. **a1** ChT at the vertical meridian; **b1** LA at the vertical meridian; **c1** SA at the vertical meridian; **d1** CVI at the vertical meridian; **a2** ChT at the horizontal meridian; **b2** LA at the horizontal meridian; **c2** SA at the horizontal meridian; **d2** CVI at the horizontal meridian. The values of LA and SA in the perifoveal regions were divided by 1.5 to eliminate the effects of inconsistent widths in each region. C0, no macular lesions; C1, tessellated fundus; C2, diffuse chorioretinal atrophy; C, fovea; I1, inferior parafovea; I2, inferior perifovea; S1, superior parafovea; S2, superior perifovea; T1, temporal parafovea; T2, temporal perifovea; N1, nasal parafovea; N2, nasal perifovea; ChT, choroidal thickness; LA, luminal area; SA, stromal area; CVI, choroidal vascular index. n = 94, 1077 and 247 eyes in C0, C1 and C2 groups, respectively. The level of significance was detected with GEE adjusted for age, AL and the correlation between both eyes of an individual; ns, not significant; ^**^P < 0.01, ^***^P < 0.001

Compared with the C1 group, the C2 group showed significant decreases in ChT, LA and SA, and increases in CVI at both vertical and horizontal meridians after adjusting for age and AL (all *P* < 0.001; Fig. 2). Compared with the C0 group, the C1 group also showed significant decreases in ChT, LA and SA, and increases in CVI at the vertical and horizontal meridians (all *P* < 0.001). Additional file 1: Table S2 presents the quantified reductions in choroidal parameters for the C1 and C2 groups in comparison to the C0 group after adjusting for age and AL. Regional analysis of choroidal parameters showed that the C2 group had lower ChT, LA, and SA across all regions than the C1 group (all *P* < 0.001; Fig. 3). Additionally, the C2 group had higher CVI across all regions except N2, I2 and S2 than the C1 group (all *P* < 0.05). Compared with the C0 group, the C1 group had lower ChT, LA, and SA across all regions (all *P* < 0.001). The C1 group had higher CVI across all regions except C region at the horizontal meridian, N2 and I2 regions than the C0 group (all *P* < 0.05).

### Associations between the presence of DCA and choroidal vascular parameters

**Fig. 4:**
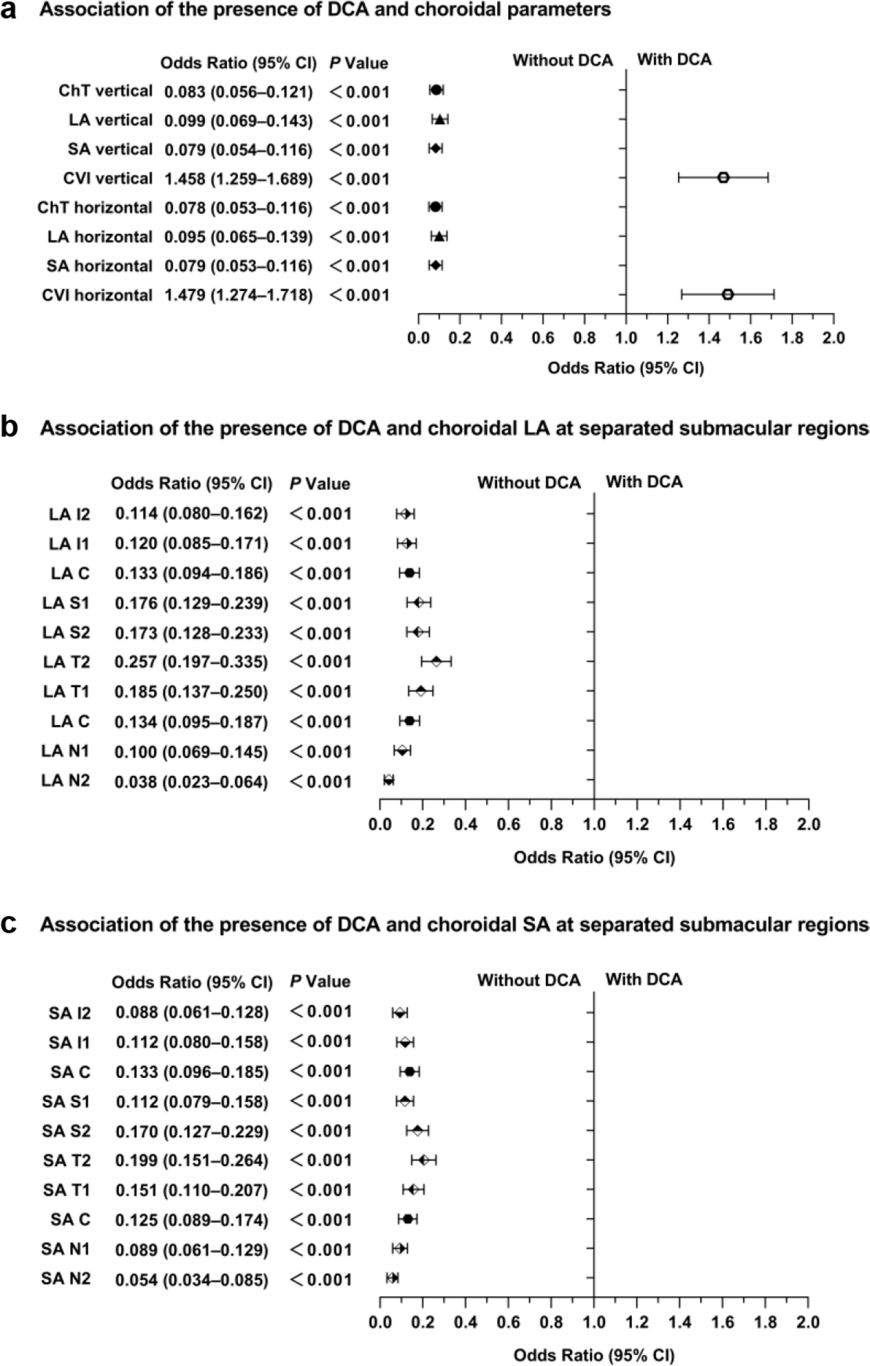
Forest plot showing risks of the presence of diffuse chorioretinal atrophy (DCA). **a** Association of the presence of DCA and choroidal parameters; **b** Association of the presence of DCA and choroidal LA at separated submacular regions; **c** Association of the presence of DCA and choroidal SA at separated submacular regions. Data were all standardized. There were 1171 eyes without DCA and 247 eyes with DCA. ChT, choroidal thickness; LA, luminal area; SA, stromal area; CVI, choroidal vascular index; CI, confidence interval. The level of significance was detected with logistic regression analysis adjusted for age, gender, AL and the correlation between both eyes of an individual

After adjusting for age, gender, AL, and the correlation between both eyes of an individual, multivariable logistic regression models revealed significant associations between the presence of DCA and choroidal vascular parameters (dependent variable: without or with DCA; Fig. 4a). Specifically, negative correlations were observed between DCA presence and both ChT [odds ratio (OR): 0.083, 95% CI: 0.056–0.121, *P* < 0.001] and LA (OR: 0.099, 95% CI: 0.069–0.143, *P* < 0.001), as well as SA (OR: 0.079, 95% CI: 0.054–0.116, *P* < 0.001). Conversely, a positive correlation was identified between DCA presence and CVI (OR: 1.458, 95% CI: 1.259–1.689, *P* < 0.001). Regional analysis showed that the most pronounced negative association with the presence of DCA was observed with LA in the N2 region (OR: 0.038, 95% CI: 0.023–0.064, *P* < 0.001; Fig. 4b–c), when compared to LA and SA in other regions. These findings imply that an increase of one standard deviation in LA within the N2 region (0.044 mm^2^) corresponds to a 96.2% decrease in the probability of DCA presence.

### Correlations between visual function and choroidal vascular parameters

Univariate linear regression analysis showed that the mean deviation (MD) was significantly and positively correlated with height (B = 0.027; *P* = 0.001), SER (B = 0.286; *P* < 0.001), ChT at the vertical meridian (B = 0.008; *P* < 0.001), LA at the vertical meridian (B = 2.286; *P* < 0.001), and SA at the vertical meridian (B = 3.176; *P* < 0.001), and inversely correlated with age (B =  − 0.009; *P* = 0.006), AL (B =  − 0.301; *P* < 0.001), and corneal curvature (B =  − 0.130; *P* = 0.005). Multivariable linear regression analysis showed that MD was positively associated with the mean LA at the vertical meridian (B = 1.512; *P* < 0.001; Table 2) after adjusting for the age, height, AL and corneal curvature. The results of the regression analysis in eyes with non-pathological myopia and pathological myopia are shown in Table 2. The results indicate that the impact of LA on visual function in eyes with pathological myopia is approximately six times greater than in eyes without pathological myopia.

**Table 2:** Correlations between MD and the mean LA at the vertical meridian

Parameters	Unstandardized coefficient		Standardized coefficient	95% CI	*P* value
	**B**	**SE**	**Beta**		
**Model 1: In all eyes**					
Constant	17.247	4.159		9.088 to 25.407	< 0.001
Age (years)	0.000	0.007	0.002	− 0.013 to 0.014	0.957
Height (cm)	0.022	0.009	0.076	0.005 to 0.039	0.013
AL (mm)	− 0.440	0.078	− 0.215	− 0.592 to − 0.288	< 0.001
Corneal curvature (D)	− 0.311	0.056	− 0.193	− 0.420 to − 0.201	< 0.001
LA_V (mm^2^)	1.512	0.324	0.147	0.877 to 2.148	< 0.001
**Model 2: In eyes with non-pathological myopia (C0 and C1)**					
Constant	8.760	4.830		− 0.719 to 18.239	0.070
Age (years)	0.010	0.007	0.043	− 0.005 to 0.024	0.181
Height (cm)	0.021	0.009	0.081	0.003 to 0.040	0.021
AL (mm)	− 0.281	0.094	− 0.130	− 0.464 to − 0.097	0.003
Corneal curvature (D)	− 0.208	0.063	− 0.136	− 0.332 to − 0.084	0.001
LA_V (mm^2^)	0.833	0.344	0.081	0.157 to 1.509	0.016
**Model 3: In eyes with pathological myopia (C2)**					
Constant	20.003	9.366		1.537 to 38.469	0.034
Age (years)	− 0.029	0.017	− 0.113	− 0.063 to 0.005	0.090
Height (cm)	0.010	0.023	0.029	− 0.036 to 0.056	0.670
AL (mm)	− 0.313	0.166	− 0.148	− 0.639 to 0.013	0.060
Corneal curvature (D)	− 0.429	0.124	− 0.252	− 0.673 to − 0.185	0.001
LA_V (mm^2^)	4.861	1.545	0.227	1.816 to 7.906	0.002

*C0* = no macular lesions; *C1* = tessellated fundus; *C2* = diffuse chorioretinal atrophy; *SE = *standard error; *CI =* confidence interval; *AL = *axial length; *LA_V = *the mean luminal area at the vertical meridian. *P* values were determined by multivariable linear regression analysis

Multivariable linear regression analysis showed that MD was positively associated with the mean SA at the vertical meridian (B = 1.956; *P* < 0.001; Additional file 1: Table S3) after adjusting for the age, height, AL and corneal curvature. The results of the regression analysis in eyes with non-pathological myopia and pathological myopia are shown in Additional file 1: Table S3. The results indicate that MD is not significantly affected by SA at the vertical meridian in eyes with non-pathological myopia. The impact of SA on visual function in eyes with pathological myopia is approximately three times greater than in all highly myopic eyes.

### Cut-off values of choroidal parameters to classify pathological myopia

**Fig. 5:**
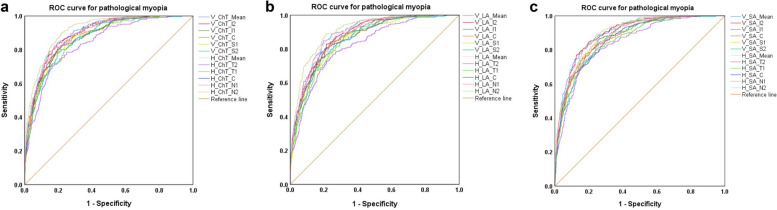
ROC curve analysis of optimal ChT, LA, and SA to diagnose pathological myopia. **a** ChT in different regions to diagnose pathological myopia; **b** LA in different regions to diagnose pathological myopia; **c** SA in different regions to diagnose pathological myopia. ROC, receiver operating characteristic; ChT, choroidal thickness; LA, luminal area; SA, stromal area; V, vertical; H, horizontal

ROC curve analysis was performed to determine the optimal ChT, LA and SA value that will aid in diagnosing pathological myopia at different locations (Fig. 5) in the overall population (Table 3). The optimal ChT to diagnose pathological myopia was 82.4 µm in the N2 region [area under the curve (AUC), 0.916; sensitivity, 89.5%; specificity, 78.5%], and 145.5 µm at the vertical meridian (AUC, 0.899; sensitivity, 84.2%; specificity, 80.4%). The optimal LA to diagnose pathological myopia was 0.076 mm^2^ in the N2 region (AUC, 0.908; sensitivity, 89.1%; specificity, 78.6%), and 0.528 mm^2^ at the vertical meridian (AUC, 0.890; sensitivity, 85.0%; specificity, 80.2%). The optimal SA to diagnose pathological myopia was 0.049 mm^2^ in the N2 region (AUC, 0.895; sensitivity, 89.1%; specificity, 75.5%), and 0.325 mm^2^ at the vertical meridian (AUC, 0.901; sensitivity, 80.6%; specificity, 83.6%).

**Table 3:** Optimal cut-off values of ChT, LA, and SA to classify pathological myopia using ROC curve analysis

Age (years)	Cut-off value	Sensitivity (%)	Specificity (%)	Youden index	AUC	SE	95% CI	*P* value
**Optimal cut-off values of ChT, LA and SA at the vertical meridian**								
All								
ChT_V (μm)	145.5	84.2	80.4	0.646	0.899	0.010	0.879 to 0.918	< 0.001
LA_V (mm^2^)	0.528	85.0	80.2	0.652	0.890	0.010	0.871 to 0.910	< 0.001
SA_V (mm^2^)	0.325	80.6	83.6	0.642	0.901	0.010	0.882 to 0.920	< 0.001
18 ≤ Age ≤ 30								
ChT_V (μm)	145.5	86.5	83.4	0.699	0.899	0.019	0.863 to 0.936	< 0.001
LA_V (mm^2^)	0.555	92.3	79.9	0.722	0.887	0.019	0.849 to 0.925	< 0.001
SA_V (mm^2^)	0.345	88.5	80.6	0.691	0.908	0.019	0.870 to 0.945	< 0.001
30 < Age ≤ 40								
ChT_V (μm)	147.5	77.3	80.1	0.574	0.863	0.019	0.825 to 0.902	< 0.001
LA_V (mm^2^)	0.528	76.1	81.5	0.576	0.860	0.020	0.822 to 0.899	< 0.001
SA_V (mm^2^)	0.354	80.7	74.6	0.553	0.860	0.020	0.822 to 0.899	< 0.001
40 < Age ≤ 50								
ChT_V (μm)	135.6	89.3	83.7	0.730	0.922	0.016	0.890 to 0.953	< 0.001
LA_V (mm^2^)	0.522	93.3	75.2	0.685	0.907	0.018	0.872 to 0.942	< 0.001
SA_V (mm^2^)	0.325	92.0	83.7	0.757	0.930	0.014	0.902 to 0.958	< 0.001
50 < Age ≤ 60								
ChT_V (μm)	129.3	84.4	92.7	0.771	0.930	0.028	0.875 to 0.985	< 0.001
LA_V (mm^2^)	0.483	84.4	90.9	0.753	0.927	0.029	0.870 to 0.984	< 0.001
SA_V (mm^2^)	0.322	87.5	81.8	0.693	0.923	0.029	0.867 to 0.980	< 0.001
**Optimal cut-off values of ChT, LA and SA in the N2 region**								
All								
ChT_N2 (μm)	82.4	89.5	78.5	0.680	0.916	0.008	0.900 to 0.933	< 0.001
LA_N2 (mm^2^)	0.076	89.1	78.6	0.677	0.908	0.009	0.890 to 0.926	< 0.001
SA_N2 (mm^2^)	0.049	89.1	75.5	0.646	0.895	0.009	0.877 to 0.914	< 0.001
18 ≤ Age ≤ 30								
ChT_N2 (μm)	84.9	96.2	78.2	0.744	0.924	0.015	0.894 to 0.954	< 0.001
LA_N2 (mm^2^)	0.076	92.3	80.6	0.729	0.917	0.017	0.884 to 0.950	< 0.001
SA_N2 (mm^2^)	0.043	88.5	83.0	0.715	0.913	0.015	0.883 to 0.943	< 0.001
30 < Age ≤ 40								
ChT_N2 (μm)	82.8	89.8	79.5	0.693	0.916	0.014	0.889 to 0.943	< 0.001
LA_N2 (mm^2^)	0.077	87.5	79.5	0.670	0.904	0.015	0.874 to 0.935	< 0.001
SA_N2 (mm^2^)	0.048	88.6	76.4	0.650	0.895	0.016	0.865 to 0.926	< 0.001
40 < Age ≤ 50								
ChT_N2 (μm)	75.1	85.3	86.3	0.716	0.920	0.016	0.889 to 0.951	< 0.001
LA_N2 (mm^2^)	0.073	90.7	78.8	0.695	0.907	0.017	0.873 to 0.941	< 0.001
SA_N2 (mm^2^)	0.043	85.3	84.4	0.697	0.909	0.017	0.876 to 0.943	< 0.001
50 < Age ≤ 60								
ChT_N2 (μm)	66.6	68.8	94.5	0.633	0.888	0.037	0.815 to 0.960	< 0.001
LA_N2 (mm^2^)	0.062	75.0	92.7	0.677	0.876	0.040	0.797 to 0.954	< 0.001
SA_N2 (mm^2^)	0.047	81.3	81.8	0.631	0.853	0.042	0.771 to 0.934	< 0.001

*ChT = *choroidal thickness; *LA = *luminal area; *SA*=Stromal area; *ROC = *receiver operating characteristic; *AUC = *area under the curve; *SE = *standard error; *CI = *confidence interval; *ChT_V = *the mean choroidal thickness at the vertical meridian; *LA_V = *the mean luminal area at the vertical meridian; *SA_V = *the mean stromal area at the vertical meridian; *ChT_N2 = *the mean choroidal thickness in the nasal perifoveal region; *LA_N2 = *the mean luminal area in the nasal perifoveal region; *SA_N2 = *the mean stromal area in the nasal perifoveal region. *P* values were determined by ROC curve analysis

The values of choroidal parameters at the vertical meridian and the N2 region had better performance in diagnosing pathological myopia for the overall participants. There were also significant differences in choroidal parameters at the vertical meridian and N2 region among different age groups after adjusting for AL (Additional file 1: Table S4). Therefore, we calculated the cut-off values for these two regions in each age group (Table 3). In patients aged ≤ 40 years, the cut-off values for ChT, LA, and SA in the N2 region exhibited higher Youden indexes. Conversely, in patients aged between 40 and 60 years, the cut-off values for ChT, LA, and SA at the vertical meridian showed higher Youden indexes.

## Discussion

This study developed an automated choroidal segmentation algorithm tailored for a highly myopic population. We delineated the changes in choroidal vasculature of early-stage myopic maculopathy and investigated the association between DCA and choroidal vasculature as well as the impact of the choroidal vasculature on visual function. We found that in addition to the previously reported risk factors such as age, AL, degree of myopia, and ChT [12, 14, 15, 18] (Additional file 1: Table S5), reductions of choroidal luminal and stromal content were associated with the presence of DCA. Furthermore, we determined the cut-off values of choroidal parameters for diagnosing pathological myopia across different age ranges.

In this study, we observed choroidal vasculature changes in highly myopic adults aged 18–60 years, with a mean age of 35.7 ± 9.9 years, mean AL of 26.96 ± 1.14 mm, and mean SER of − 8.37 ± 2.05 D. We found that along with progressive thinning of the whole choroid, loss of choroidal vascular and stromal components occurred with the aggravation of myopic maculopathy, with greater reductions from no macular lesions stage (C0) to tessellated fundus stage (C1) than from the C1 stage to the DCA stage (C2). These findings are consistent with those of Wang et al. [22] and Zhao et al. [23]. Regional analysis of choroidal parameters showed that eyes with C2 showed the most pronounced reduction in the foveal region than other regions when compared to eyes with C0. These observations align with the outcomes reported earlier [16]. Furthermore, during the progression from C0 to C2, a global increase in CVI was observed, showing that the reduction in stroma occurred faster than the reduction in choroidal vascular volume. Wang et al. found that compared to simple high myopia (mean AL, 27.02 ± 1.07 mm), pathological myopia (mean AL, 29.57 ± 1.59 mm) showed a significant reduction in both LA and SA, with a notable decrease in the CVI [24], indicating that the reduction in choroidal vascular volume was more rapid in pathological myopia. Taken together, there seems to be a decline in the protective effect of the extravascular stroma on the blood vessels preceding the onset of C2, leading to an accelerated decrease in vascular volume during disease progression, which needs to be proven in mechanistic studies. It was reported that increased CVI may indicate vasodilation and vascular congestion in active diseases such as wet age-related macular degeneration [25] and central serous chorioretinopathy [26, 27]. Unlike previous studies, CVI may have different meanings in such a chronic progressive disease. Regional analysis of CVI showed that from C0 to C2, the reduction rate of choroidal vascular volume relative to the stroma was faster in the N2 and I2 regions compared to other regions, suggesting that the yellow-white appearance of the fundus in these regions at the stage of DCA can be attributed to severe choroidal vessels atrophy. Luo et al. studied the registration between indocyanine green angiography (ICGA) and multi-color scanning laser (MCSL) imaging fundus images of high myopia [28]. Their images showed that the yellow-white areas of the chorioretinal atrophy in MCSL presented with fluorescence defects on ICGA, supporting the above inference.

Differences in BCVA results showed a notable decline in visual acuity in pathological myopia. Furthermore, compared with non-pathological myopia, the impact of choroidal luminal components on MD in pathological myopia was approximately six times greater. This suggests that reduced choroidal perfusion significantly affected visual function in eyes with DCA. Therefore, interventions aimed at enhancing choroidal perfusion should ideally commence during the non-pathological myopia stage to mitigate visual function deterioration. In comparison to non-pathological myopia, the effect of changes in choroidal stromal components on MD was significantly more pronounced in pathological myopia, suggesting that the choroidal stromal components were also intervenable targets. We reported visual field impairment in non-pathological myopia (MD − 3.45 ± 2.20 dB, PSD 2.31 ± 1.59 dB, and VFI 96.43 ± 4.61%), which is consistent with an earlier report by Lin et al. [29]. Compared with our findings, the MD and VFI reported by Lin et al. were higher, indicative of relatively better visual function. This discrepancy could stem from variations in the composition of the study populations. Specifically, Lin’s study included 400 eyes with C0 and 900 eyes with C1. In contrast, our study consisted of 94 and 1077 eyes in these respective categories.

We identified the threshold values for ChT and vascular parameters that aid in the diagnosis of pathological myopia across four age groups. For individuals aged 40 years and younger, the discriminative power of ChT, LA and SA within the N2 region was higher (AUC ≥ 0.895). In contrast, for those older than 40 years, the discriminative capacity was more pronounced at the vertical meridian (AUC ≥ 0.907). This distinction could likely be due to the more severe PPA in older individuals, which may compromise the ability of choroidal parameters in the N2 region to differentiate pathological conditions effectively. This study determined an optimal ChT threshold of 84.9 µm in the N2 region for diagnosing pathological myopia in the 18–30 age group, with a sensitivity of 96.2% and specificity of 78.2%, and an AUC of 0.924. Fang et al.'s research identified an optimal ChT cut-off value of 56.5 µm in the nasal region for diagnosing PDCA in individuals younger than 20 years, achieving a sensitivity of 89.5%, specificity of 87.5%, and AUC of 0.9 [16]. It should be noted that Fang’s study included individuals aged 3−20 years with high myopia of over − 8 D. On the other hand, our study cohort included individuals aged 18−30 and we defined high myopia to be − 6 D, and thus could contribute to the varying thresholds being reported.

Excessive choroidal thinning is often accompanied by remnants of large choroidal vessels leading to the formation of retinal pigment epithelium (RPE) humps [30]. In the study of Marchese et al., the incidence of RPE humps was 50.8%, which affected older patients (62.5 ± 1.3 years) and those with extremely thin choroids (foveal ChT, 38 ± 27.7 µm). However, RPE humps were only observed in 9 eyes (0.6%) in our study. The lower prevalence may be due to participants in the whole cohort being relatively younger (35.7 ± 9.9 years), and only a few exhibiting extremely thinner choroids (macular ChT, 169.0 ± 67.3 µm). Among these 9 eyes, 2 eyes had localized coarse choroidal vessels forming the RPE humps. The other 7 eyes had very thin choroids, with only partial large vessels remaining. The choroid in these 7 eyes was too thin for the algorithm to recognize correctly. Additionally, the RPE between the RPE humps was often misidentified as choroidal stroma, resulting in a thicker stroma. We discovered this segmentation error during our preliminary checks and used manual segmentation methods to circumvent this issue. Excluding these 9 eyes for ROC curve analysis, the cut-off value of ChT for diagnosing pathological myopia increased by 0.6 µm compared to before exclusion, while the cut-off values of LA and SA remained unchanged (Additional file 1: Table S6). Therefore, this condition has little influence on the results.

In our study, 3 eyes exhibited myopic tractional maculopathy, characterized by inner or outer foveoschisis. Other tractional lesions such as lamellar holes, macular holes, and retinal detachment were not seen in our study. Cases with severe foveoschisis and macular holes who have undergone surgical treatment have been excluded during the initial screening. The current number of cases was insufficient to study the correlation between myopic tractional maculopathy and choroidal structure and perfusion. Previous studies have reported changes in choriocapillaris (CC) perfusion in highly myopic individuals with myopic traction maculopathy [31, 32]. Wang et al. analyzed quantitative CC findings (flow void number, total flow void area, and average flow void area) at each stage of myopic traction maculopathy and reported that the macular ChT and CC flow had no significant correlation with tractional degrees after adjusting for age [31]. Quiroz-Reyes et al. found that there were no significant preoperative differences in CVI and CC flow area between 18 healthy highly myopic eyes (matched for sex and age) and 46 eyes with myopic traction maculopathy [32]. No significant differences were found in CVI or CC flow among different stages of myopic traction maculopathy. However, the postoperative CVI and CC flow of eyes with myopic traction maculopathy were significantly lower than those of the control eyes, providing evidence of choroidal perfusion changes after vitrectomy surgery [32]. These studies suggest that the mechanism of tractional lesions may not be related to choroidal structure or perfusion, but vitrectomy does indeed affect postoperative choroidal perfusion.

The several strengths of our study include firstly, the development of an automatic choroidal segmentation algorithm utilizing deep learning. This was specifically designed for the highly myopic population that elucidated the changes in choroidal vascular and stromal components. Secondly, our study defined the correlation between DCA and both choroidal vascular volume and extravascular stroma in a large-scale study of highly myopic individuals. Early interventions aimed at improving choroidal perfusion and stabilizing the extravascular stroma, could potentially slow down or prevent the progression of DCA. Lastly, it identified cut-off values of choroidal parameters to diagnose pathological myopia at various ages, offering a valuable reference for clinical diagnosis and intervention. The limitations of this study include the cross-sectional design that did not allow for the assessment of the temporal evolution between changes in the vasculature and the development of myopic maculopathy. Longitudinal studies are needed to explore this relationship further. Next, the reliability of visual field test results may be compromised due to the examination's limited duration, allowing for only a single reliable measurement, which could impact result stability. Addtionally, biomechanical alterations of the sclera can cause localized eyeball deformation and vision impairment. Gong et al.’s study, using fundus pulsation optical coherence elastography, revealed a significant decrease in scleral rigidity in pathologic myopia in humans aged between 60–80 years old with a mean spherical equivalent of – 13.5 D [33]. However, the presence of posterior staphyloma was not evaluated in the current study due to the limited view presented in non-wide-field OCT equipment. Lastly, CC perfusion was suggested to be correlated with DCA [34, 35], but we did not evaluate CC flow area in this study. As we are currently developing an automatic measurement algorithm for the CC flow area suitable for highly myopic eyes, only subsequent studies will include an evaluation of CC perfusion.

## Conclusions

Leveraging SS-OCT and the deep learning algorithm, this study advanced the comprehension of the choroidal vasculature in those with early-stage myopic maculopathy. It illuminated the critical role of choroidal perfusion and stromal components in the etiology of DCA and the deterioration of visual function. Moreover, it established threshold values for choroidal parameters in diagnosing pathological myopia across four age groups, offering valuable reference ranges for clinical diagnosis and management.

### Supplementary Information

Additional file 1: Table S1. Inclusion and exclusion criteria of the Wenzhou High Myopia Cohort Study. Table S2. Changes of choroidal parameters in eyes with C1 and C2 compared with C0. Table S3. Correlations between MD and the mean SA at the vertical meridian. Table S4. Effect of age grouping on choroidal parameters. Table S5. The well-known risk factors for the presence and progression of DCA reported in the literature. Table S6. Optimal cut-off values to classify pathological myopia.

## Data Availability

The data used and analyzed in this study are available from the corresponding author upon reasonable request.

## Abbreviations

DCA: Diffuse chorioretinal atrophy
SER: Spherical equivalent refractive
AL: Axial length
BCVA: Best-corrected visual acuity
SBP: Systolic blood pressure
DBP: Diastolic blood pressure
CCT: Central corneal thickness
ACD: Anterior chamber depth
LT: Lens thickness
IOP: Intraocular pressure
SS-OCT: Swept-source optical coherence tomography
ChT: Choroidal thickness
LA: Luminal area
SA: Stromal area
TCA: Total choroidal area
CVI: Choroidal vascular index
RPE: Retinal pigment epithelium
ROC: Receiver operating characteristic
AUC: Area under the curve
VFI: Visual field index
MD: Mean deviation
PSD: Pattern standard deviation
PPA: Parapapillary atrophy
CC: Choriocapillaris
