# Supplementary material for: Choroidal vascular changes in early-stage myopic maculopathy from deep learning choroidal analysis: a hospital-based SS-OCT study
Source: Eye Vis (Lond). 2024 Aug 6;11:32. doi: 10.1186/s40662-024-00398-x (PMC11301841; doi:10.1186/s40662-024-00398-x)
Supplement: Supplementary file 1 — Additional file 1: Table S1. Inclusion and exclusion criteria of the Wenzhou High Myopia Cohort Study. Table S2. Changes of choroidal parameters in eyes with C1 and C2 compared with C0. Table S3. Correlations between MD and the mean SA at the vertical meridian. Table S4. Effect of age grouping on choroidal parameters. Table S5. The well-known risk factors for the presence and progression of DCA reported in the literature. Table S6. Optimal cut-off values to classify pathological myopia. [file 40662_2024_398_MOESM1_ESM.zip › 40662_2024_398_MOESM1_ESM_ESM.docx]

### Additional files

**Additional file 1: Table S1.** Inclusion and exclusion criteria of the Wenzhou High Myopia Cohort Study.

| **Inclusion criteria** |
| --- |
| Age between 10 and 60 years inclusive. |
| SER ≤ −6.00 D or AL ≥ 26.5 mm in either eye and astigmatism ≤ 2.50 D. |
| **Exclusion criteria** |
| Unable to provide informed consent or unable to return for scheduled protocol visits. |
| Early onset high myopia and secondary myopia, such as a history of retinopathy of prematurity, or syndromic myopia such as Stickler or Marfan syndrome. |
| Having had keratorefractive surgery for myopia or any intraocular surgery affecting refractive status. |
| Wearing orthokeratology lenses within 1 month or wearing rigid gas permeable contact lenses within 2 weeks. |
| Having autoimmunity disease such as systemic lupus erythematosus, rheumatoid arthritis; or having severe health problems precluding follow-up such as heart disease, kidney disease, or terminal cancer. |
| Having significant ocular media opacity preventing fundus examinations. |
