# Supplementary material for: Choroidal vascular changes in early-stage myopic maculopathy from deep learning choroidal analysis: a hospital-based SS-OCT study
Source: Eye Vis (Lond). 2024 Aug 6;11:32. doi: 10.1186/s40662-024-00398-x (PMC11301841; doi:10.1186/s40662-024-00398-x)
Supplement: Supplementary file 1 — Additional file 1: Table S1. Inclusion and exclusion criteria of the Wenzhou High Myopia Cohort Study. Table S2. Changes of choroidal parameters in eyes with C1 and C2 compared with C0. Table S3. Correlations between MD and the mean SA at the vertical meridian. Table S4. Effect of age grouping on choroidal parameters. Table S5. The well-known risk factors for the presence and progression of DCA reported in the literature. Table S6. Optimal cut-off values to classify pathological myopia. [file 40662_2024_398_MOESM1_ESM.zip › 40662_2024_398_MOESM2_ESM_ESM.docx]

**Additional file 1: Table S2.** Changes of choroidal parameters in eyes with C1 and C2 compared with C0.

| **Parameters** | **Unstandardized coefficient** | | **Standardized coefficient** | **95% CI** | ***P* value** |
| --- | --- | --- | --- | --- | --- |
|  | **B** | **SE** | **Beta** |  |  |
| **Model 1: Dependent** **variable: LA_V** | | | | | |
| Constant | 2.324 | 0.120 |  | 2.089 to 2.558 | < 0.001 |
| Age (years) | −0.001 | 0.000 | −0.052 | −0.002 to 0.000 | 0.012 |
| AL (mm) | −0.049 | 0.004 | −0.245 | −0.057 to −0.040 | < 0.001 |
| TF (C1) | −0.294 | 0.019 | −0.553 | −0.332 to −0.257 | < 0.001 |
| DCA (C2) | −0.495 | 0.023 | −0.826 | −0.540 to −0.451 | < 0.001 |
| **Model 2: Dependent variable: SA_V** | | | | | |
| Constant | 1.527 | 0.079 |  | 1.371 to 1.682 | < 0.001 |
| Age (years) | 0.000 | 0.000 | −0.011 | −0.001 to 0.000 | 0.604 |
| AL (mm) | −0.033 | 0.003 | −0.243 | −0.038 to −0.027 | < 0.001 |
| TF (C1) | −0.212 | 0.013 | −0.591 | −0.237 to −0.187 | < 0.001 |
| DCA (C2) | −0.354 | 0.015 | −0.875 | −0.383 to −0.324 | < 0.001 |
| **Model 3: Dependent variable: ChT_V** | | | | | |
| Constant | 641.821 | 32.690 |  | 577.695 to 705.947 | < 0.001 |
| Age (years) | −0.228 | 0.131 | −0.036 | −0.485 to 0.029 | 0.082 |
| AL (mm) | −13.556 | 1.216 | −0.247 | −15.941 to −11.171 | < 0.001 |
| TF (C1) | −84.383 | 5.222 | −0.573 | −94.625 to −74.140 | < 0.001 |
| DCA (C2) | −141.552 | 6.201 | −0.853 | −153.716 to −129.388 | < 0.001 |
| The classification of myopic maculopathy was set up with dummy variables and the control group was C0.  C0 = no macular lesions; C1 = tessellated fundus (TF); C2 = diffuse chorioretinal atrophy (DCA); SE = standard error; CI = confidence interval; AL = axial length; LA_V = the mean luminal area at the vertical meridian; SA_V = the mean stromal area at the vertical meridian; ChT_V = the mean choroidal thickness at the vertical meridian. *P* values were determined by multivariable linear regression analysis. | | | | | |
