# Supplementary material for: Choroidal vascular changes in early-stage myopic maculopathy from deep learning choroidal analysis: a hospital-based SS-OCT study
Source: Eye Vis (Lond). 2024 Aug 6;11:32. doi: 10.1186/s40662-024-00398-x (PMC11301841; doi:10.1186/s40662-024-00398-x)
Supplement: Supplementary file 1 — Additional file 1: Table S1. Inclusion and exclusion criteria of the Wenzhou High Myopia Cohort Study. Table S2. Changes of choroidal parameters in eyes with C1 and C2 compared with C0. Table S3. Correlations between MD and the mean SA at the vertical meridian. Table S4. Effect of age grouping on choroidal parameters. Table S5. The well-known risk factors for the presence and progression of DCA reported in the literature. Table S6. Optimal cut-off values to classify pathological myopia. [file 40662_2024_398_MOESM1_ESM.zip › 40662_2024_398_MOESM3_ESM_ESM.docx]

**Additional file 1: Table S3.** Correlations between MD and the mean SA at the vertical meridian.

| **Parameters** | **Unstandardized** **coefficient** | | **Standardized coefficient** | **95% CI** | ***P* value** |
| --- | --- | --- | --- | --- | --- |
|  | **B** | **SE** | **Beta** |  |  |
| **Model 1: In all eyes** | | | | | |
| Constant | 17.644 | 4.180 |  | 9.444 to 25.844 | < 0.001 |
| Age (years) | −0.001 | 0.007 | −0.005 | −0.015 to 0.012 | 0.872 |
| Height (cm) | −0.460 | 0.078 | −0.224 | −0.612 to −0.307 | < 0.001 |
| AL (mm) | 0.024 | 0.009 | 0.083 | 0.006 to 0.041 | 0.007 |
| Corneal curvature (D) | −0.310 | 0.056 | −0.193 | −0.419 to −0.200 | < 0.001 |
| SA_V (mm^2^) | 1.956 | 0.481 | 0.128 | 1.012 to 2.899 | < 0.001 |
| **Model 2: In eyes with non-pathological myopia (C0 and C1)** | | | | | |
| Constant | 9.144 | 4.847 |  | −0.368 to 18.657 | 0.060 |
| Age (years) | 0.009 | 0.007 | 0.040 | −0.005 to 0.024 | 0.215 |
| Height (cm) | −0.298 | 0.094 | −0.139 | −0.483 to −0.114 | 0.002 |
| AL (mm) | 0.023 | 0.009 | 0.087 | 0.005 to 0.041 | 0.013 |
| Corneal curvature (D) | −0.207 | 0.064 | −0.135 | −0.332 to −0.083 | 0.001 |
| SA_V (mm^2^) | 0.918 | 0.514 | 0.060 | −0.090 to 1.926 | 0.074 |
| **Model 3: In eyes with pathological myopia (C2)** | | | | | |
| Constant | 20.939 | 9.392 |  | 2.421 to 39.456 | 0.027 |
| Age (years) | −0.035 | 0.017 | −0.132 | −0.068 to −0.001 | 0.045 |
| Height (cm) | −0.339 | 0.165 | −0.161 | −0.665 to −0.013 | 0.041 |
| AL (mm) | 0.014 | 0.023 | 0.040 | −0.032 to 0.059 | 0.561 |
| Corneal curvature (D) | −0.435 | 0.124 | −0.256 | −0.680 to −0.191 | 0.001 |
| SA_V (mm^2^) | 6.742 | 2.355 | 0.202 | 2.099 to 11.385 | 0.005 |
| MD = mean deviation; C0 = no macular lesions; C1 = tessellated fundus; C2 = diffuse chorioretinal atrophy; SE = standard error; CI = confidence interval; AL = axial length; SA_V = the mean stromal area at the vertical meridian. *P* values were determined by multivariable linear regression analysis. | | | | | |
