# Supplementary material for: Choroidal vascular changes in early-stage myopic maculopathy from deep learning choroidal analysis: a hospital-based SS-OCT study
Source: Eye Vis (Lond). 2024 Aug 6;11:32. doi: 10.1186/s40662-024-00398-x (PMC11301841; doi:10.1186/s40662-024-00398-x)
Supplement: Supplementary file 1 — Additional file 1: Table S1. Inclusion and exclusion criteria of the Wenzhou High Myopia Cohort Study. Table S2. Changes of choroidal parameters in eyes with C1 and C2 compared with C0. Table S3. Correlations between MD and the mean SA at the vertical meridian. Table S4. Effect of age grouping on choroidal parameters. Table S5. The well-known risk factors for the presence and progression of DCA reported in the literature. Table S6. Optimal cut-off values to classify pathological myopia. [file 40662_2024_398_MOESM1_ESM.zip › 40662_2024_398_MOESM4_ESM_ESM.docx]

**Additional file 1: Table S4.** Effect of age grouping on choroidal parameters.

| **Parameter** | **B** | **SE** | **95% CI** | ***P* value** |
| --- | --- | --- | --- | --- |
| **Dependent variable：ChT_V** | | |  |  |
| Intercept | 812.820 | 31.5942 | 750.897 to 874.744 | < 0.001 |
| Age group |  |  |  | 0.002 |
| Age group 4 | −17.900 | 6.816 | −31.259 to −4.541 | 0.009 |
| Age group 3 | −13.073 | 3.8759 | −20.669 to −5.476 | 0.001 |
| Age group 2 | −4.928 | 3.6472 | −12.076 to 2.221 | 0.177 |
| Age group 1 | Ref | - | - | - |
| AL | −23.266 | 1.1756 | −25.570 to −20.962 | < 0.001 |
| **Dependent variable：LA_V** | |  |  |  |
| Intercept | 2.910 | 0.1138 | 2.687 to 3.133 | < 0.001 |
| Age group |  |  |  | < 0.001 |
| Age group 4 | −0.080 | 0.024 | −0.127 to −0.033 | 0.001 |
| Age group 3 | −0.051 | 0.0141 | −0.079 to −0.023 | < 0.001 |
| Age group 2 | −0.022 | 0.0131 | −0.048 to 0.004 | 0.092 |
| Age group 1 | Ref | - | - | - |
| AL | −0.083 | 0.0042 | −0.091 to −0.074 | < 0.001 |
| **Dependent variable：SA_V** | |  |  |  |
| Intercept | 1.966 | 0.0785 | 1.813 to 2.120 | < 0.001 |
| Age group |  |  |  | 0.019 |
| Age group 4 | −0.028 | 0.0173 | −0.061 to 0.006 | 0.111 |
| Age group 3 | −0.027 | 0.0094 | −0.046 to −0.009 | 0.003 |
| Age group 2 | −0.008 | 0.009 | −0.025 to 0.010 | 0.403 |
| Age group 1 | Ref | - | - | - |
| AL | −0.057 | 0.0029 | −0.063 to −0.051 | < 0.001 |
| **Dependent variable：ChT_N2** | |  |  |  |
| Intercept | 521.166 | 24.6259 | 472.900 to 569.432 | < 0.001 |
| Age group |  |  |  | 0.047 |
| Age group 4 | −6.764 | 5.4906 | −17.526 to 3.997 | 0.218 |
| Age group 3 | −8.156 | 2.9444 | −13.926 to −2.385 | 0.006 |
| Age group 2 | −4.254 | 2.7934 | −9.729 to 1.221 | 0.128 |
| Age group 1 | Ref | - | - | - |
| AL | −15.135 | 0.9172 | −16.932 to −13.337 | < 0.001 |
| **Dependent variable：LA_N2** | |  |  |  |
| Intercept | 0.485 | 0.0237 | 0.439 to 0.532 | < 0.001 |
| Age group |  |  |  | 0.003 |
| Age group 4 | −0.010 | 0.0052 | −0.020 to 0.001 | 0.064 |
| Age group 3 | −0.010 | 0.0028 | −0.015 to −0.004 | < 0.001 |
| Age group 2 | −0.005 | 0.0027 | −0.010 to 0.000 | 0.058 |
| Age group 1 | Ref | - | - | - |
| AL | −0.014 | 0.0009 | −0.016 to −0.012 | < 0.001 |
| **Dependent variable：SA_N2** | |  |  |  |
| Intercept | 0.297 | 0.0143 | 0.269 to 0.324 | < 0.001 |
| Age group |  |  |  | 0.602 |
| Age group 4 | 0.000 | 0.0032 | −0.007 to 0.006 | 0.880 |
| Age group 3 | −0.002 | 0.0017 | −0.006 to 0.001 | 0.185 |
| Age group 2 | −0.001 | 0.0016 | −0.005 to 0.002 | 0.406 |
| Age group 1 | Ref | - | - | - |
| AL | −0.009 | 0.0005 | −0.010 to −0.008 | < 0.001 |
| Age group 1: 18 ≤ age ≤ 30 years; Age group 2: 30 < age ≤ 40 years; Age group 3: 40 < age ≤ 50 years; Age group 4: 50 < age ≤ 60 years.  SE = standard error; CI = confidence interval; AL = axial length; ChT_V = the mean choroidal thickness at the vertical meridian; LA_V = the mean luminal area at the vertical meridian; SA_V = the mean stromal area at the vertical meridian; ChT_N2 = the mean choroidal thickness in the nasal perifoveal region; LA_N2 = the mean luminal area in the nasal perifoveal region; SA_N2 = the mean stromal area in the nasal perifoveal region. *P* values were determined by GEE. | | | | |
