# Supplementary material for: Choroidal vascular changes in early-stage myopic maculopathy from deep learning choroidal analysis: a hospital-based SS-OCT study
Source: Eye Vis (Lond). 2024 Aug 6;11:32. doi: 10.1186/s40662-024-00398-x (PMC11301841; doi:10.1186/s40662-024-00398-x)
Supplement: Supplementary file 1 — Additional file 1: Table S1. Inclusion and exclusion criteria of the Wenzhou High Myopia Cohort Study. Table S2. Changes of choroidal parameters in eyes with C1 and C2 compared with C0. Table S3. Correlations between MD and the mean SA at the vertical meridian. Table S4. Effect of age grouping on choroidal parameters. Table S5. The well-known risk factors for the presence and progression of DCA reported in the literature. Table S6. Optimal cut-off values to classify pathological myopia. [file 40662_2024_398_MOESM1_ESM.zip › 40662_2024_398_MOESM5_ESM_ESM.docx]

**Additional file 1: Table S5.** The well-known risk factors for the presence and progression of DCA reported in the literature.

| **Reference** | **Patient (high myopia)** | **Study type** | **Dependent variable** | **Related factors** | **OR (95% CI)** |
| --- | --- | --- | --- | --- | --- |
| Chen et al. 2011 [14] | 337 patients (aged 8–88 years) | Cross-sectional | Presence of DCA | Older age | 1.05 (1.01–1.09) |
|  |  |  |  | Lower degree of myopia | 0.58 (0.46–0.72) |
| Guo et al. 2021 [15] | 274 children (ages < 18 years) | Longitudinal | Incident of PDCA | Lower degree of myopia | 0.70 (0.54–0.92) |
|  |  |  |  | Greater gamma zone enlargement | 8.28 (1.33–51.7) |
| Deng et al. 2023 [18] | 579 children (aged 4–18 years) | Cross-sectional | Presence of DCA | Thicker macular ChT | 0.942 (0.926–0.959) |
| Li et al. 2021 [12] | 484 patients (aged 7–70 years) | Longitudinal | Progression of DCA | Longer AL | 1.89 |
|  |  |  |  | Presence of baseline DCA | 1.62 |
|  |  |  |  | Baseline DCA closer to the fovea | 4.31 |
| DCA = diffuse chorioretinal atrophy; OR = odds ratio; CI = confidence interval; PDCA = peripapillary diffuse chorioretinal atrophy; ChT = choroidal thickness; AL = axial length. | | | | | |
