# Supplementary material for: Choroidal vascular changes in early-stage myopic maculopathy from deep learning choroidal analysis: a hospital-based SS-OCT study
Source: Eye Vis (Lond). 2024 Aug 6;11:32. doi: 10.1186/s40662-024-00398-x (PMC11301841; doi:10.1186/s40662-024-00398-x)
Supplement: Supplementary file 1 — Additional file 1: Table S1. Inclusion and exclusion criteria of the Wenzhou High Myopia Cohort Study. Table S2. Changes of choroidal parameters in eyes with C1 and C2 compared with C0. Table S3. Correlations between MD and the mean SA at the vertical meridian. Table S4. Effect of age grouping on choroidal parameters. Table S5. The well-known risk factors for the presence and progression of DCA reported in the literature. Table S6. Optimal cut-off values to classify pathological myopia. [file 40662_2024_398_MOESM1_ESM.zip › 40662_2024_398_MOESM6_ESM_ESM.docx]

**Additional file 1: Table S6.** Optimal cut-off values to classify pathological myopia (excluding the 9 eyes with RPE humps).

| **Parameters** | **Cut-off value** | **Sensitivity (%)** | **Specificity (%)** | **Youden  index** | **AUC** | **SE** | **95% CI** | ***P* value** |
| --- | --- | --- | --- | --- | --- | --- | --- | --- |
| **Optimal cut-off values of ChT, LA and SA at the vertical meridian** | | | | | | | | |
| All |  |  |  |  |  |  |  |  |
| ChT_V (μm) | 146.1 | 84.1 | 80.1 | 0.642 | 0.896 | 0.01 | 0.876 to 0.915 | < 0.001 |
| LA_V (mm^2^) | 0.528 | 84.5 | 80.3 | 0.648 | 0.888 | 0.01 | 0.867 to 0.908 | < 0.001 |
| SA_V (mm^2^) | 0.325 | 79.9 | 83.6 | 0.635 | 0.898 | 0.01 | 0.878 to 0.917 | < 0.001 |
| **Optimal cut-off values of ChT, LA and SA in the N2 region** | | | | | | | | |
| All |  |  |  |  |  |  |  |  |
| ChT_N2 (μm) | 83.0 | 90.0 | 77.7 | 0.677 | 0.913 | 0.01 | 0.896 to 0.930 | < 0.001 |
| LA_N2 (mm^2^) | 0.076 | 88.7 | 78.5 | 0.672 | 0.905 | 0.01 | 0.886 to 0.923 | < 0.001 |
| SA_N2 (mm^2^) | 0.049 | 88.7 | 75.5 | 0.642 | 0.892 | 0.01 | 0.873 to 0.912 | < 0.001 |

RPE = retinal pigment epithelium; ChT = choroidal thickness; LA = luminal area; SA = stromal area; AUC = area under the curve; SE = standard error; CI = confidence interval; ChT_V = the mean choroidal thickness at the vertical meridian; LA_V = the mean luminal area at the vertical meridian; SA_V = the mean stromal area at the vertical meridian; ChT_N2 = the mean choroidal thickness in the nasal perifoveal region; LA_N2 = the mean luminal area in the nasal perifoveal region; SA_N2 = the mean stromal area in the nasal perifoveal region. *P* values were determined by ROC curve analy
